# Physicians as Speakers

**Published:** 1900-10

**Authors:** 


A Monthly Review of Medicine and Surgery.

*EDITOR:*

WILLIAM WARREN POTTER, M. D.

All communications, whether of a literary or business nature, books for review and exchanges should be addressed to the editor: 284 Franklin Street, Buffalo, N. Y.

Vol. XL.—LVI. OCTOBER, 1900. No. 3.

### PHYSICIANS AS SPEAKERS.

THE multiplication of medical societies, general and special, has developed a speaking capacity of late among physicians that formerly was quite unknown. Papers are read that are to be printed and these must be discussed and the discussions published if the value of the contribution is to be developed into its greatest usefulness. Physicians are, speaking generally, but indifferent debaters, hence the pith and kernel of their remarks often fail of their intended effect. No one is more cognizant of the imperfections of physicians as speakers than the medical reporter who is brought face to face with the facts.

Mr. William Whitford, of Chicago, writes on this subject in the *Jour. of the American Medical Association*, September 22, 1900. He offers many suggestions of value and interest to physicians in regard to public speaking, which all who participate in medical discussions will do well to heed. As Mr. Whitford is the official stenographer for the American Medical Association, and of several state and special medical societies, his article is based on an extensive and varied experience in reporting the utterances of doctors. He says that the communication of thoughts by means of spoken language is an art that cannot be acquired to any great degree of perfection except by long and continued practice. However distinct may be a physician's voice, however vivid his conceptions, he is often conscious that the phraseology at his command, particularly in public speaking, is inadequate to do them justice. He seeks in vain the words he needs, and strives ineffectually to devise forms of expression which shall faithfully portray his thought.

The pitch, intensity and amplitude of a speaker's voice have much to do with the work of the reporter, in that they will either greatly expedite his work or render it extremely difficult. A physician, in addressing a convention in a large hall, may pitch his voice so low that perhaps only one-half of the members can hear distinctly what he says, and it is largely guess work for the other half to grasp his meaning. The acoustics of a hall may be perfect, but if the speaker's voice is improperly pitched, he will be imperfectly heard; and, in such instances, incorrectly reported. The physician who takes cognisance of acoustics and pitches his voice in the proper key, if he articulates well, can be heard in the remotest nook or corner of a large convention hall without apparently any unusual effort. A clear, distinct, far-reaching voice is undoubtedly a natural gift, although physicians, as speakers, can do much by training to acquire an agreeable tone. In reporting loud, excitable speakers the stenographer meets with difficulty at times in catching the exact words on account of deficient enunciation on the part of the speaker or muffled syllables. Bright, Gladstone, Phillips, Clay and other great orators understood the secret of how to pitch the voice so as to make it audible to everybody in a large hall. If the acoustic properties of a hall are good, the voice pitched in the right key will carry; if those properties are bad, the shouted words will be heard no better than if they were whispered.

A medium pitch should be the basis of speech. From it one may rise or fall, according to intellectual and emotional requirements. Height and depth are necessary. Beecher said, "What a speaker most needs is to strengthen his ordinary conversational voice, without giving it a hard, firm quality, that is, without destroying its flexibility and power of adaptation to every mood." It is a fine art to be able to lower one's pitch. Some scream on to the end during an animated discussion stopping from sheer exhaustion; others spasmodically fall to a low note, but immediately forget themselves and run up to the same pitch, vociferating till out of breath.

The acoustic properties of most auditoriums are imperfect; but these defects generally have a physical cause which admits of being guarded against by the adaptation of the speaker's position and tone. Monotony of a low-pitched voice exerts a sporific influence over an audience, which no strength of thought nor beauty of language can wholly counteract and, if there be regularly recurring minor notes, the most startling expressions lose their power. Even to those who do not sleep the sounds bear no sense.

The prime object of a speaker should be to make himself understood, and to this end sense should never be sacrificed to sound. A speaker should never use more force than necessary. If requested to speak louder, he should beware of raising the pitch of his voice; by a slight increase of volume on the same key he can make anyone, whose organs of hearing are not defective, hear distinctly. The groundwork of good speaking is the tone of lively conversation. The essence of distinctness is a clear, crisp articulation. With some speakers the vowels absolutely drown the consonants, which have thus no opportunity of asserting themselves, and the result is that the hearers have but a vague conception of the words that are uttered.

Continuing he says of delivery that the first duty of a physician in addressing an audience is to make himself heard. If he speaks very rapidly his hearers will miss words here and there, and he fails to produce the effect intended. The speaker should be full of his subject and impressed with its importance. He should speak deliberately, enunciate distinctly, and in a natural voice. He should express his thoughts with clearness and force. His peroration should be sharp, a clear summary of established propositions, forced home perhaps by an impressive illustration.

Mr. Whitford further says that one of the facts disclosed by reporting physicians for the last sixteen years, is the marked tendency towards greater simplicity of expression. Involved sentences and the use of big words to say little things are not tolerated as they once were. Conciseness and condensation, as elements of style, have largely taken the place of classicism. It is not so much stateliness as incisiveness that is sought. The axiom of the modern speaker appears to be that to talk effectively one must speak tersely.

A common error among young physicians, in discussing papers read before medical societies, is a tendency to diffuseness, which may be defined as a copious use of words so arranged as to create the suspicion that a thought is somewhere concealed among them. The youthful physician in debate feels it necessary to describe ordinary things in an extraordinary way, and strives to dignify commonplace thoughts by clothing them in fulsome rhetoric. The result is a painful incongruity between the thoughts and their apparel. For instance, a young physician instead of saying that a man was thrown sideways from his carriage, breaking his leg and putting his ankle out of joint, said that "the patient was projected transversely from his vehicle, fracturing the tibia and fibula and luxating the tibio-tarsal articulation." Mr. Whitford gives several additional examples. No one is better situated to note the disastrous effect of the inapt and ineffective use of words than the reporter. Pope expressed the matter in a few words when he said:

> "Words are like leaves, and where they most abound,
> Much fruit of sense beneath is rarely found."

The first principle of strong oral composition demands the employment of as few words as will clearly express the thought. This rule, carefully followed, will soon eliminate redundancy, circumlocution and all kindred evils that weaken the style of many otherwise good speakers.

As one of the greatest elements of success in good speaking, Mr. Whitford mentions careful and thorough preparation and a clear idea of the subject to be discussed. No physician can expect to sway his hearers unless he is a perfect master of the subject in hand. In growing earnest, impressive, or in reaching for a climax, it is not necessary for a speaker to rant or roar. Much energy is wasted by professors in lecturing to medical students in this way that might be advantageously saved. To be energetic and eloquent, it is not necessary to declaim boisterously.

*Physicians and lawyers compared.*—The art of speaking well is not confined to statesmen, jurists and clergymen. The medical profession, like the ministerial and legal professions, has orators within its ranks. The experienced physician strives to use the simplest and most expressive words. The author of the paper we are quoting has had the pleasure of reporting the utterances of several eminent lawyers from time to time, and, to speak candidly, he says that they were not the superiors in any sense of many of the physicians whose impromptu speeches he has reported. He mentions two or three striking examples of the extemporaneous type of speakers in the medical profession. Of Dr. Nicholas Senn he says: "He is a fluent, forcible, and impressive speaker. He has an excellent command of language. There is a richness in his diction, a copiousness, ease, and variety in his expression which are rarely surpassed by the best extemporaneous speakers. The arrangement of his sentences in debate seem never to have been studied, yet every word falls into its proper place. He displays his ability as a debater to best advantage when under a heavy fire."

Of Dr. Charles A. L. Reed, of Cincinnati, Ohio, the President of the American Medical Association, he says: "He is a splendid example of the extemporaneous type of speaker. As a debater he is well equipped. He is noted for his elegant diction, his scholarly references, and the ornateness of his phraseology. His well rounded sentences are marvels of construction. No matter how sudden the summons, or what the subject, a speech from him is always interesting. His rhetoric is faultless, his delivery graceful and easy."

He regards Dr. Joseph M. Mathews, of Louisville, as "an easy, graceful, fascinating, polished speaker, whose style is notable for its simplicity. He can adorn any subject."

As examples of good extemporaneous speakers in the medical profession, the author mentions some thirty representative physicians of the East, West, North, and South, and closes his paper with a classification embracing thirty-seven types of speakers—good, bad and indifferent. From this imperfect résumé it will be observed that the article is well worth reading and we hope all participants in medical society work will study it.

